# Bioinformatics Analysis of Candidate Genes and Pathways Related to Hepatocellular Carcinoma in China: A Study Based on Public Databases

**DOI:** 10.3389/pore.2021.588532

**Published:** 2021-03-26

**Authors:** Peng Zhang, Jing Feng, Xue Wu, Weike Chu, Yilian Zhang, Ping Li

**Affiliations:** ^1^ School of Graduates, Tianjin Medical University, Tianjin, China; ^2^ Department of Hepatology, Tianjin Second People’s Hospital, Tianjin, China; ^3^ Tianjin Research Institute of Liver Diseases, Tianjin, China

**Keywords:** China, hepatocellular carcinoma, differentially expressed genes, hub genes, cell cycle, bioinformatics analysis

## Abstract

**Background and Objective:** Hepatocellular carcinoma (HCC) is a highly aggressive malignant tumor of the digestive system worldwide. Chronic hepatitis B virus (HBV) infection and aflatoxin exposure are predominant causes of HCC in China, whereas hepatitis C virus (HCV) infection and alcohol intake are likely the main risk factors in other countries. It is an unmet need to recognize the underlying molecular mechanisms of HCC in China.

**Methods:** In this study, microarray datasets (GSE84005, GSE84402, GSE101685, and GSE115018) derived from Gene Expression Omnibus (GEO) database were analyzed to obtain the common differentially expressed genes (DEGs) by R software. Moreover, the gene ontology (GO) functional annotation and Kyoto Encyclopedia of Genes and Genomes (KEGG) pathway analysis were performed by using Database for Annotation, Visualization and Integrated Discovery (DAVID). Furthermore, the protein-protein interaction (PPI) network was constructed, and hub genes were identified by the Search Tool for the Retrieval of Interacting Genes (STRING) and Cytoscape, respectively. The hub genes were verified using Gene Expression Profiling Interactive Analysis (GEPIA), UALCAN, and Kaplan-Meier Plotter online databases were performed on the TCGA HCC dataset. Moreover, the Human Protein Atlas (HPA) database was used to verify candidate genes’ protein expression levels.

**Results:** A total of 293 common DEGs were screened, including 103 up-regulated genes and 190 down-regulated genes. Moreover, GO analysis implied that common DEGs were mainly involved in the oxidation-reduction process, cytosol, and protein binding. KEGG pathway enrichment analysis presented that common DEGs were mainly enriched in metabolic pathways, complement and coagulation cascades, cell cycle, p53 signaling pathway, and tryptophan metabolism. In the PPI network, three subnetworks with high scores were detected using the Molecular Complex Detection (MCODE) plugin. The top 10 hub genes identified were *CDK1*, *CCNB1*, *AURKA*, *CCNA2*, *KIF11*, *BUB1B*, *TOP2A*, *TPX2*, *HMMR* and *CDC45*. The other public databases confirmed that high expression of the aforementioned genes related to poor overall survival among patients with HCC.

**Conclusion:** This study primarily identified candidate genes and pathways involved in the underlying mechanisms of Chinese HCC, which is supposed to provide new targets for the diagnosis and treatment of HCC in China.

## Introduction

Liver cancer is the sixth most common cancer and the fourth leading cause of cancer-related death worldwide, posing a significant challenge to public health [1]. Hepatocellular carcinoma (HCC) accounts for approximately 90% of all primary liver cancers [2]. Genetic abnormalities, cellular context, and external environment play essential roles in the development of HCC. Interestingly, the main risk factors of HCC vary by region. For example, because of diverse traditional dietary habits and diseases susceptibility, the predominant causes of HCC are chronic hepatitis B virus (HBV) infection and aflatoxin exposure in China, whereas hepatitis C virus (HCV) infection and alcohol intake are likely the main risk factors in other countries [1, 3]. It has been reported that more than 120 million people carried hepatitis B surface antigen (HBsAg), and approximately 54% of HCCs are attributed to HBV infection in China [2, 4]. In addition, Non-alcoholic fatty liver disease (NAFLD) is increasingly a cause of cirrhosis and hepatocellular carcinoma in China with the popularity of the sedentary lifestyle and fast food culture brought by the expansion of urbanization in recent years [5]. Since HCC has its unique genetic and environmental background in China, it is vital to investigate the molecular mechanisms involved in the occurrence, progression, and metastasis of HCC from China to improve diagnostic and therapeutic strategies. Therefore, it is an unmet need to identify relevant genes and signaling pathways involved in the pathophysiology of HCC to achieve effective diagnosis and treatment in the early stage of HCC in China.

In recent years, bioinformatics analysis based on microarrays and high-throughput sequencing technologies has been widely used to identifying differentially expressed genes (DEGs) and functional pathways related to the occurrence and development of various diseases [6, 7]. However, given the high false-positive rates, it is difficult to obtain reliable results from independent microarrays or high-throughput sequencing analysis. Fortunately, to get reliable results, integrated bioinformatics analyses have been developed to perform a large-scale analysis of cross-platform microarrays or high-throughput data.

In this study, microarray datasets GSE84005 [8], GSE84402 [9], GSE101685 (unpublished), and GSE115018 [10] about HCC in China derived from Gene Expression Omnibus (GEO) database were downloaded and analyzed to obtain DEGs between HCC tissues and normal tissue. Subsequently, Gene Ontology (GO) functional enrichment analysis and Kyoto Encyclopedia of Genes and Genomes (KEGG) pathway enrichment analysis was performed. Furthermore, protein-protein interaction (PPI) networks were constructed to identify subnetworks and hub genes. Above all, this work is supposed to identify potential candidate genes and provide new therapeutic targets for the advancement of HCC from China.

## Materials and Methods

### Microarray Data

With the continuous innovation of high-throughput technology, the results of some outdated datasets are relatively inaccurate. In order to eliminate the errors caused by the imbalance of high-throughput sequencing technology, the microarray datasets were searched from the Gene Expression Omnibus (GEO) database (https://www.ncbi.nlm.nih.gov/geo/) from January 1st, 2016 to October 30th, 2019 using the following keywords: “hepatocellular carcinoma or HCC” (study keyword), “*Homo sapiens*” (organism), “Expression profiling by array” (study type) and “tissue” (attribute name). After a systematic review (Supplementary Figure S1), four gene expression profiles about HCC in China (GSE84005, GSE84402, GSE101685, and GSE115018) were collected for further analysis. GSE84402 and GSE101685 were based on the platforms of GPL570 [HG-U133_Plus_2] Affymetrix Human Genome U133 Plus 2.0 Array, while GSE84005 and GSE115018 were GPL5175 [HuEx-1_0-st] Affymetrix Human Exon 1.0 ST Array and GPL20115 Agilent-067406 Human CBC lncRNA + mRNA microarray V4.0 respectively. lncRNA data of GSE115018 were not analyzed in this study. GSE84005 (Beijing), GSE84402 (Shanghai), GSE101685 (Taipei), and GSE115018 (Nanning) contain 38, 14, 8, and 12 cases of normal tissues and 38, 14, 24, and 12 cases of HCC tissues from Chinese patients separately.

### Screening for Common Differentially Expressed Genes

The GEOquery package was used to download the series matrix of the four databases in R (v3.6.1). The gene expression data were subjected to quantile normalization by the Linear Models for Microarray Data (limma) package before analysis. The limma package was used to identify DEGs between normal tissues and HCC tissues in each dataset, which is based on unpaired t-test. The DEGs were identified according to the thresholds that adjusted *p* value (adj.P.Val) < 0.05 and *|*log fold change (FC)*|* > 1. The DEGs were visualized using the pheatmap and the ggplot2 packages. The DEGs intersection of four datasets was used to obtain the common DEGs of HCC in China by Venny 2.1.0 (https://bioinfogp.cnb.csic.es/tools/venny/index.html.).

### Gene Ontology and Kyoto Encyclopedia of Genes and Genomes Pathway Enrichment Analyses

The Database for Annotation, Visualization, and Integrated Discovery (DAVID 6.8, https://david.ncifcrf.gov/) is a shared database for gene enrichment and functional annotation analysis, which integrates biodata and analytical tools to provide a systematic and comprehensive annotation of biological functions for large-scale lists of genes or proteins [11]. In order to preliminary understand biological functions and pathway enrichment of the common DEGs, GO and KEGG pathway enrichment analysis was performed by using DAVID online tool. The results of GO and KEGG pathway enrichment analysis TXT files were downloaded and visualized using the R. *p* < 0.05 was considered statistically significant.

### Protein-Protein Interaction Network Construction and Subnetwork Analysis

The Search Tool for the Retrieval of Interacting Genes (STRING v11, https://string-db.org/) is designed to construct a critical assessment and integration of protein-protein interaction (PPI) network [12]. To understand the interactional correlation of the common DEGs, a PPI network was established by STRING, and then the results of the PPI network TSV file were downloaded and visualized by Cytoscape (3.7.2) that is a public bioinformatics software [13]. Furthermore, the Molecular Complex Detection (MCODE) plugin [14] was also applied to select the significant subnetworks from the PPI network (degree cutoff ≥ 2, node score cutoff ≥ 0.2, K-core ≥ 2, and max depth = 100, score ≥ 5). Moreover, the KEGG analyses for genes in subnetworks were used to investigate their potential biological functions using DAVID. *p* < 0.05 was considered statistically significant.

### Hub Genes Identification and Prognosis Analysis

In the PPI network, hub genes, the top ten genes with the highest degree, were identified using the CytoHubba plugin [15]. The Gene Expression Profiling Interactive Analysis (GEPIA, http://gepia.cancer-pku.cn) was used to evaluate mRNA expression of hub genes in The Cancer Genome Atlas (TCGA) database [16]. Besides, the Human Protein Atlas (HPA, https://www.proteinatlas.org/) database was used to verify the protein expression level of candidate genes in HCC tissues [17]. Furthermore, the association between selected genes and the prognosis of HCC was analyzed using UALCAN (http://ualcan.path.uab.edu) online tool on TCGA HCC cases [18]. According to the upper quartile cutoff levels of gene expression, the HCC cases in the TCGA database cases are separated into high-expression and low/medium-expression groups in survival analysis. *p* < 0.05 was considered statistically significant. To further clarify the influence of the region, environment, and living habits on survival outcomes, we conducted a subgroup analysis of HCC patients by ethnicity using Kaplan-Meier Plotter (https://kmplot.com/), whose primary purpose is a meta-analysis based on discovery and validation of survival biomarkers [19]. There were 364 HCC patients with available clinical data, including 184 White/Caucasian and 158 Asian. *p* < 0.05 was considered statistically significant. The methods above are summarized in Supplementary Figure S2.

## Results

### Identification of Common DEGs of HCC From China

**FIGURE 1:**
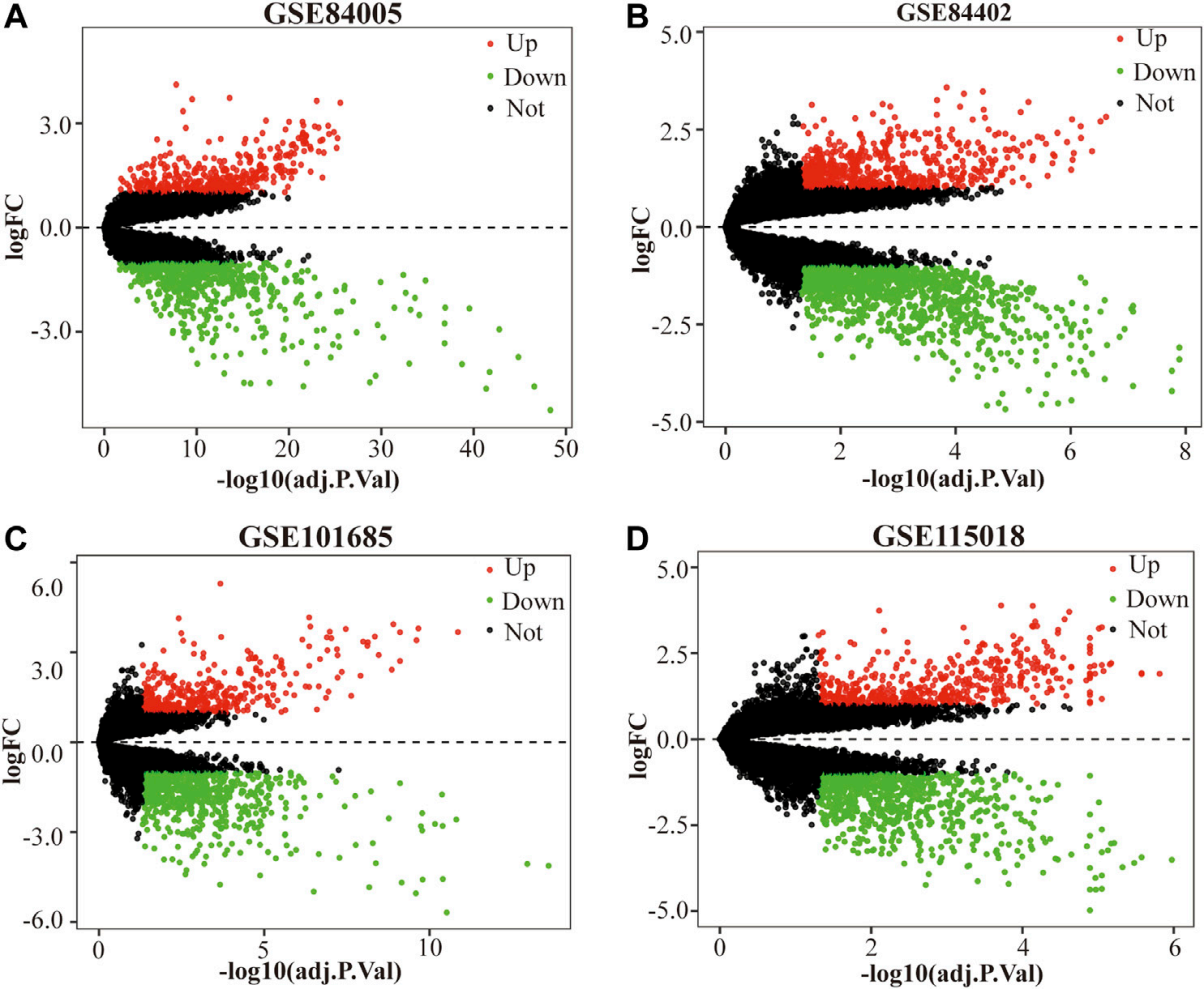
The DEGs between HCC tissue samples and normal tissue samples in each dataset. **(A)** GSE84005, **(B)** GSE84402, **(C)** GSE101685 and **(D)** GSE115018. The red dots represent the upregulated genes based on adjusted *p* value (adj.P.Val) < 0.05 and log fold change (FC) > 1, the green dots represent the downregulated genes based on adj.P.Val <0.05 and log FC < 1; the black spots represent genes with no significant difference. DEGs, differentially expressed genes. HCC, hepatocellular carcinoma.

**FIGURE 2:**
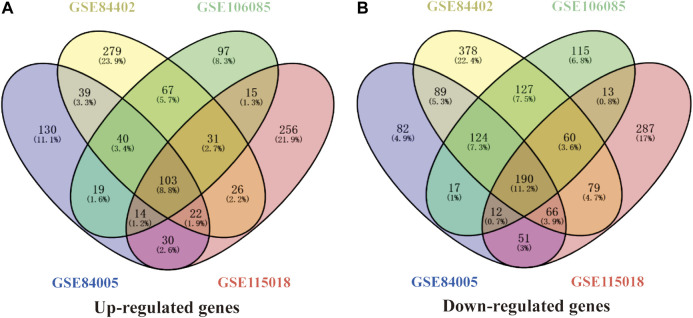
A total of 293 common DEGs were identified from the four HCC datasets in China. **(A)** and **(B)** represents 103 common up-regulated genes and 190 common down-regulated genes identified from GSE84005, GSE84402, GSE101685, and GSE115018 datasets. DEGs, differentially expressed genes.

Four gene expression matrices were normalized before analysis, and the results are shown in Supplementary Figure S3. In addition, there was 1028 (397 up-regulated genes, 631 down-regulated genes), 1720 (607 up-regulated genes, 1113 down-regulated genes), 1044 (386 up-regulated genes, 658 down-regulated genes) and 1282 (497 up-regulated genes, 785 down-regulated genes) screened from GSE84005, GSE84402, GSE101685 and GSE115018 respectively in Table 1, Figures 1A–D and Supplementary Figure S4. Furthermore, through the DEGs intersection of four datasets using Venny 2.1.0, a total of 293 common DEGs were identified, including 103 up-regulated genes and 190 down-regulated genes (Figures 2A,B, Supplementary Tables S1, S2).

**TABLE 1:** Information of DEGs identified from each dataset from China.

GEO	Sample	City	Up-regulated genes	Down-regulated genes	Total of DEGs
GSE84005	HCC	Beijing	397	631	1028
GSE84402	HCC	Shanghai	607	1113	1720
GSE101685	HCC	Taipei	386	658	1044
GSE115018	HCC	Nanning	497	785	1282

DEGs, differentially expressed genes; GEO, gene expression omnibus; HCC, hepatocellular carcinoma.

### Gene Ontology and Kyoto Encyclopedia of Genes and Genomes Pathway Enrichment Analyses

**FIGURE 3:**
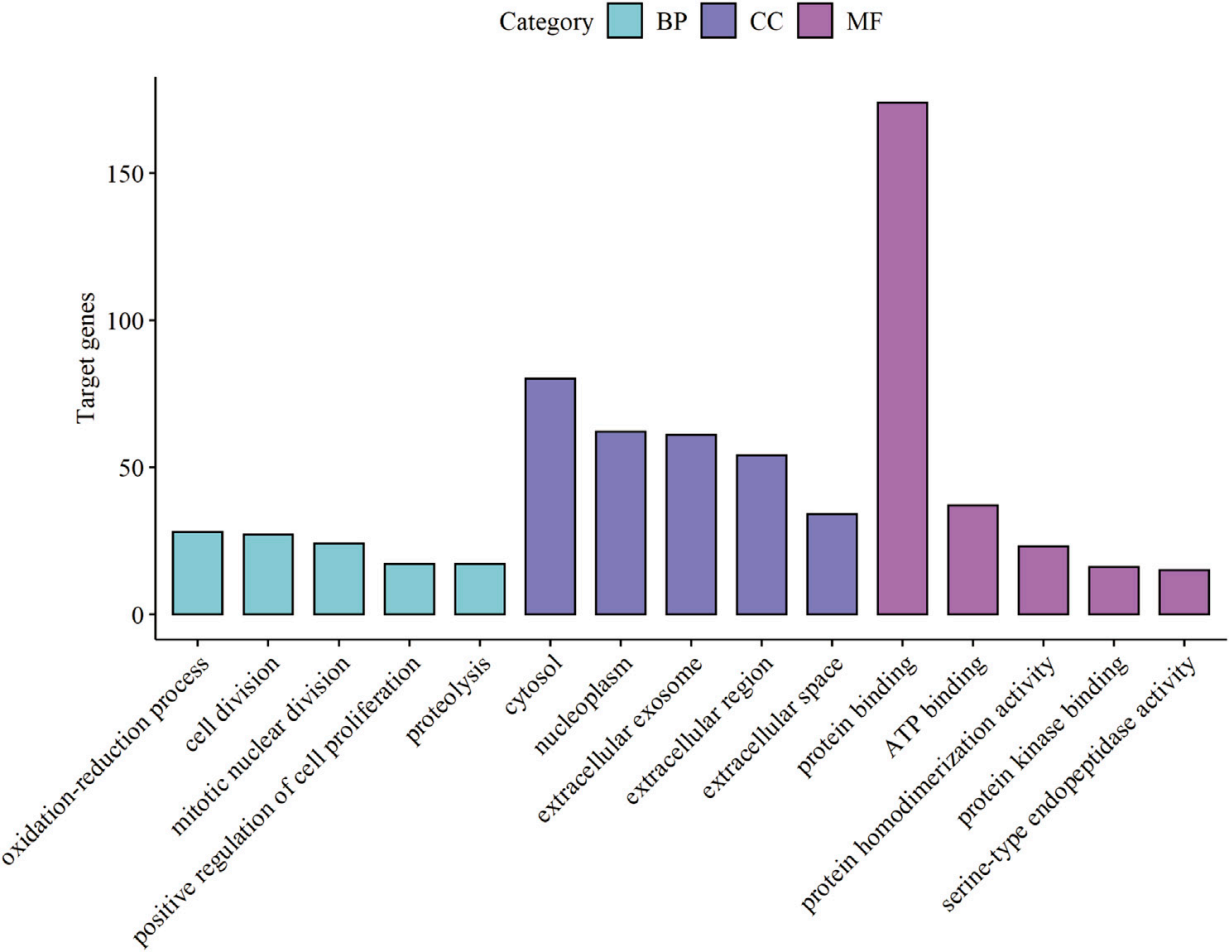
Top 15 GO enrichment terms of the common DEGs. Biological Process (BP), Cellular Component (CC), and Molecular Function (MF) category are represented by sky blue, dark blue, and purple bars, respectively. GO, Gene Ontology. DEGs, differentially expressed genes.

**FIGURE 4:**
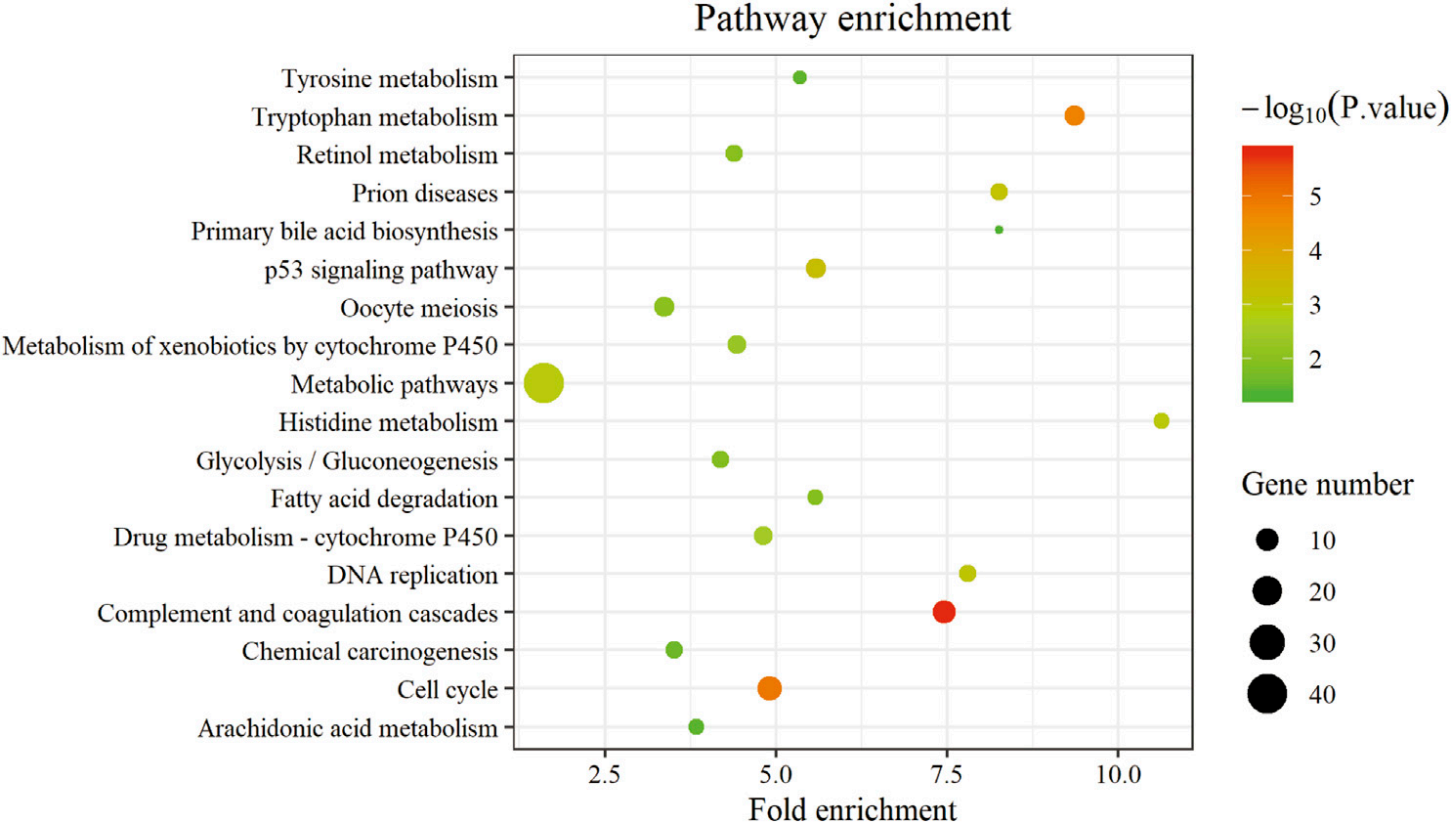
The KEGG pathway of the common DEGs. The abscissa represents the fold enrichment; the ordinate represents the pathway terms. The size of bubble represents gene number enriched in this pathway, and the color of bubble represents statistical difference. KEGG, Kyoto Encyclopedia of Genes and Genomes. DEGs, differentially expressed genes.

GO analysis consists of three functional parts, including biological process (BP), cellular component (CC), and molecular function (MF). As shown in Figure 3 and Supplementary Table S3, the results of GO analysis indicated that the common DEGs were enriched in the BP, including oxidation-reduction process, cell division, mitotic nuclear division, positive regulation of cell proliferation, and proteolysis. For the CC, the common DEGs were principally enriched in the cytosol, nucleoplasm, extracellular exosome, extracellular region, and extracellular space. As for the MF, the common DEGs were mainly enriched in protein binding, ATP binding, protein homodimerization activity, protein kinase binding, and serine-type endopeptidase activity. Additionally, the KEGG pathway enrichment analysis results revealed that the common DEGs were particularly enriched in metabolic pathways, complement and coagulation cascades, cell cycle, p53 signaling pathway, and tryptophan metabolism shown in Figure 4 and Supplementary Table S4.

### Protein-Protein Interaction Network Construction and Subnetwork Analysis

**FIGURE 5:**
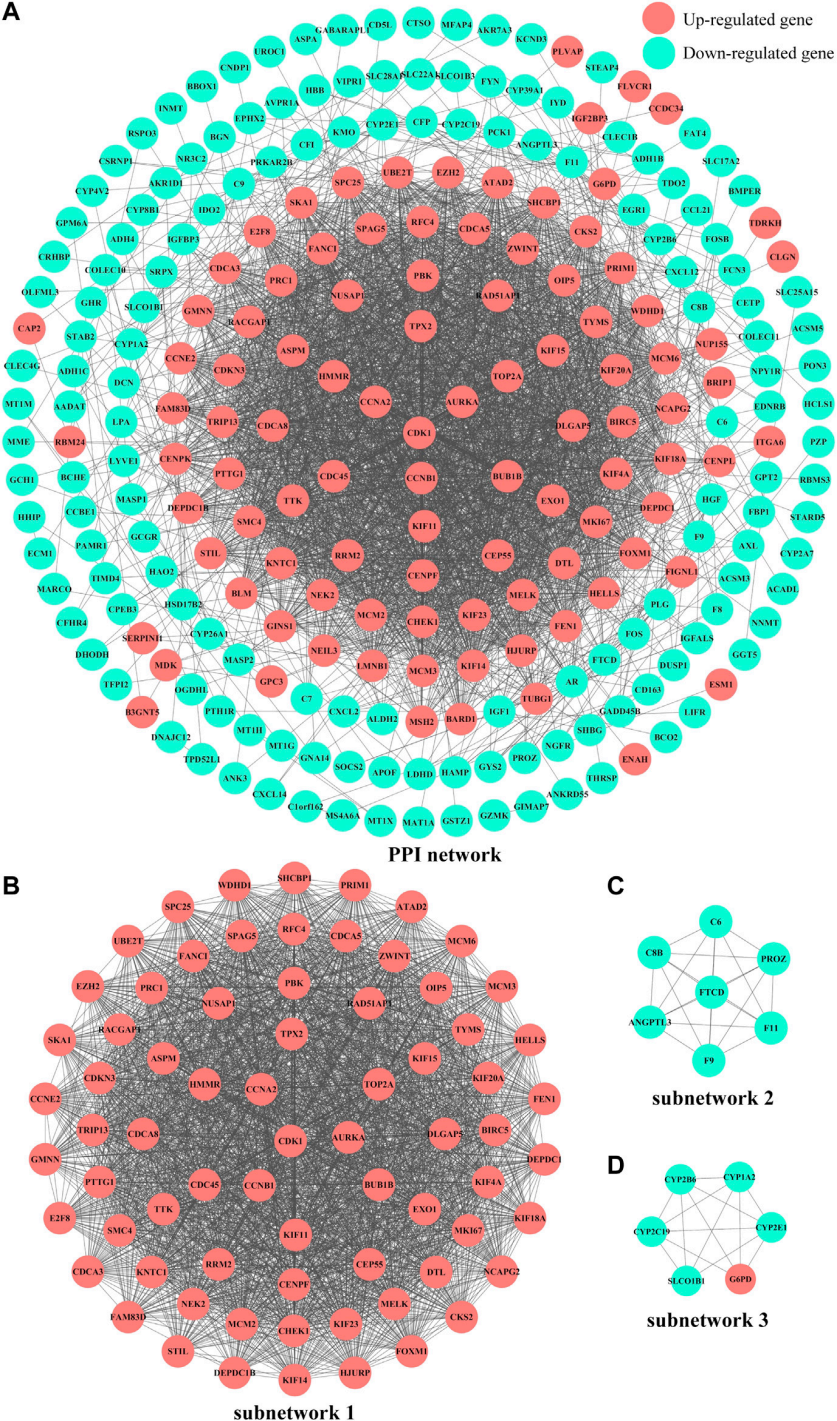
PPI network diagrams of common DEGs and subnetworks from the Cytoscape software. **(A)**PPI network of common DEGs. **(B)**subnetwork 1, MCODE score = 65.493. **(C)**subnetwork 2, MCODE score = 7. **(D)**subnetwork 3, MCODE score = 5.6. Red nodes and blue nodes represent upregulated genes and downregulated genes, respectively. PPI, protein-protein interaction. DEGs, differentially expressed genes.

The PPI network was initially constructed by importing the 293 common DEGs from four microarray datasets about HCC in China into the STRING online database (Supplementary Figure S5). Next, the network diagram was presented by using Cytoscape, which was composed of 253 nodes and 3113 edges, as shown in Figure 5A. Furthermore, the three most significant subnetworks (Figures 5B–D, Supplementary Table S5) of the PPI network were selected. The results of KEGG analysis showed that the genes in subnetwork 1 were particularly enriched in cell cycle, DNA replication, p53 signaling pathway, oocyte meiosis, viral carcinogenesis, and progesterone-mediated oocyte maturation; subnetwork 2 was principally enriched in complement and coagulation cascades and prion diseases; and the subnetwork 3 was mainly enriched in metabolic pathways, drug metabolism - cytochrome P450, linoleic acid metabolism, arachidonic acid metabolism, and metabolism of xenobiotics by cytochrome P450 and retinol metabolism, as shown in Supplementary Table S6 and Supplementary Figure S6.

### Hub Genes Identification and Prognosis Analysis

**FIGURE 6:**
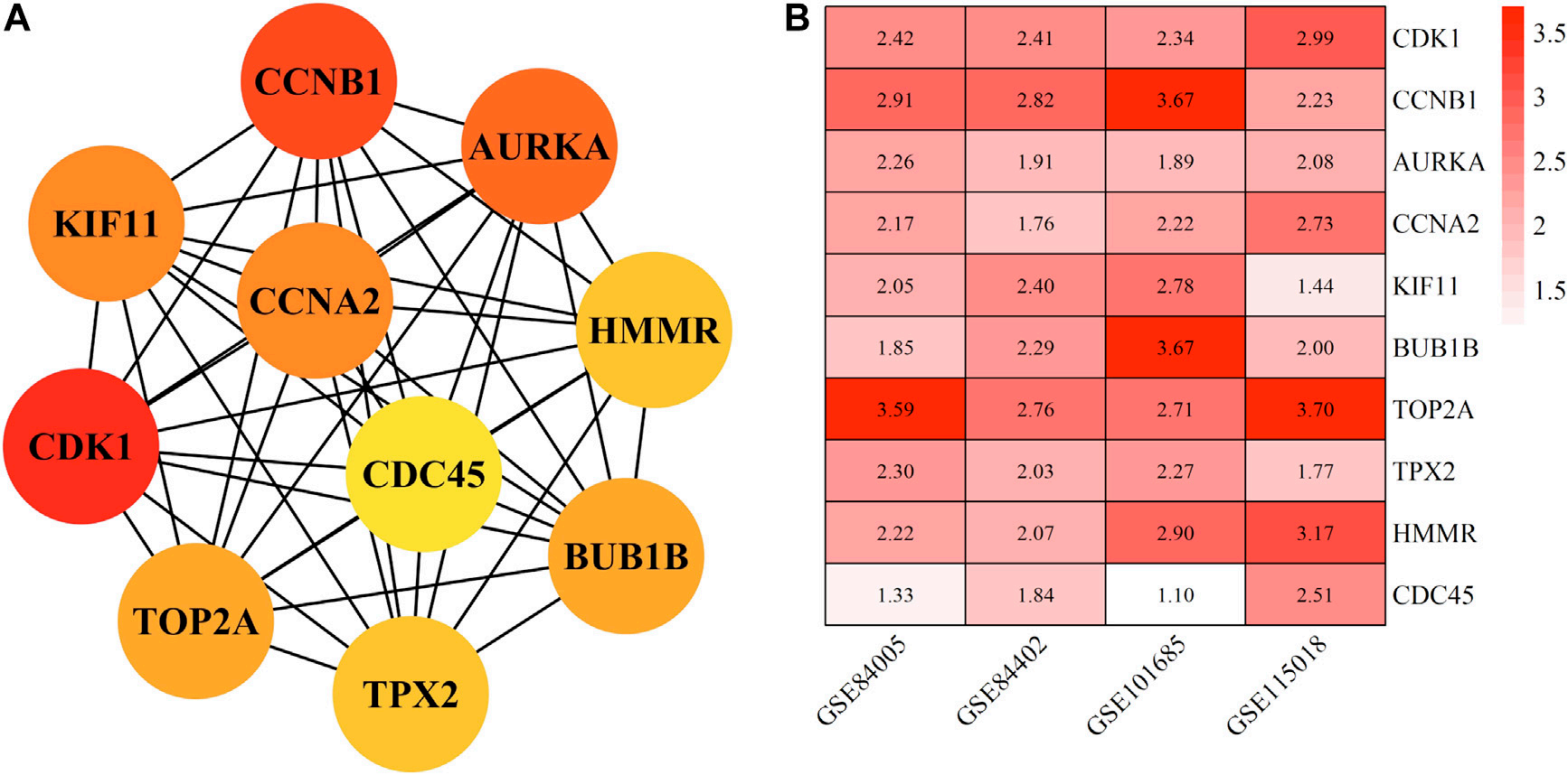
The top ten hub genes of HCC in China. **(A)** shows the interaction network interaction between hub genes. **(B)** shows differential expression of ten hub genes in different datasets. The abscissa represents the GEO accessions; the ordinate represents the gene name, the value in the box represents the log FC value. HCC, hepatocellular carcinoma. FC, fold change.

**FIGURE 7:**
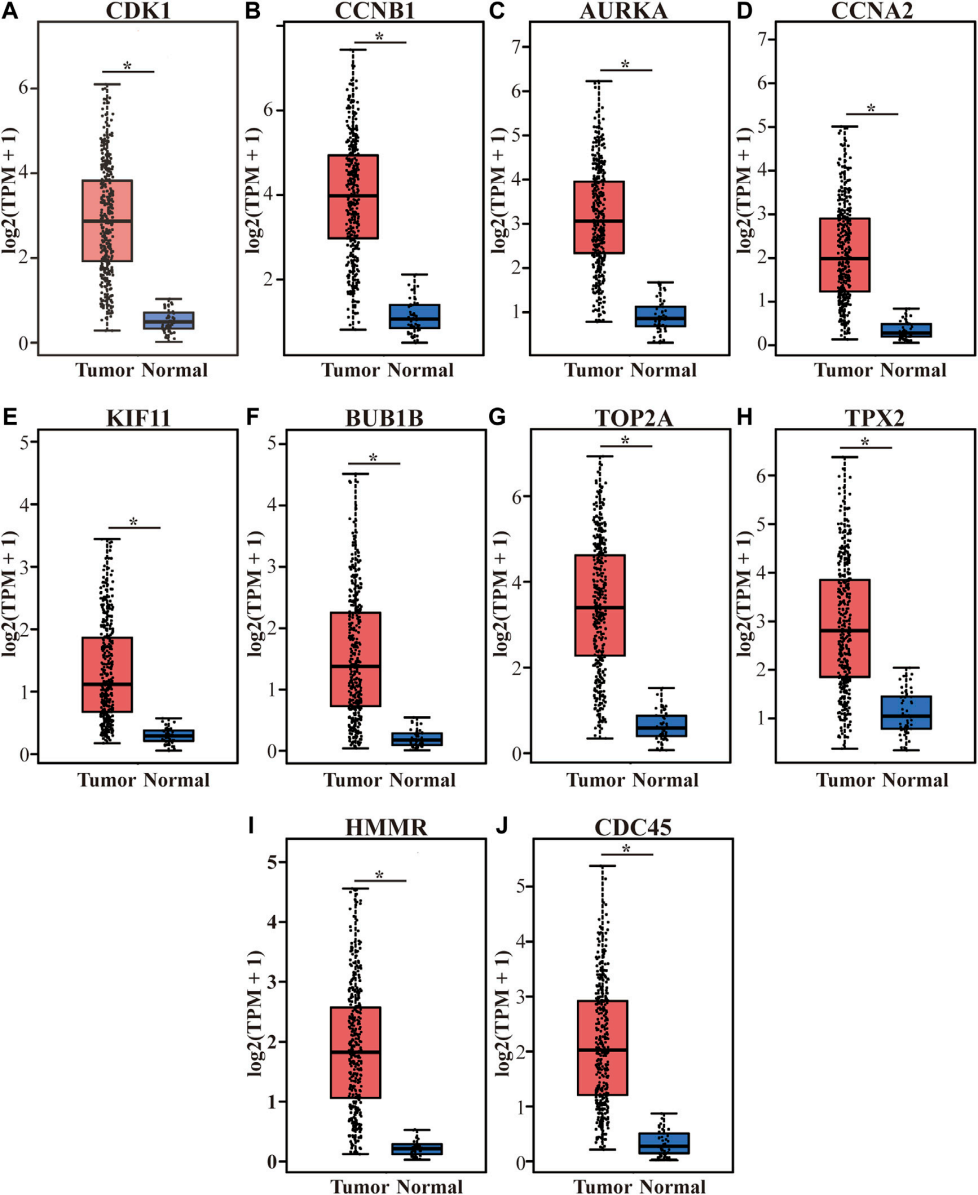
Differential expression of the top 10 hub genes on the TCGA LIHC dataset in the GEPIA database. **(A–J)** represent *CDK1*, *CCNB1*, *AURKA*, *CCNA2*, *KIF11*, *BUB1B*, *TOP2A*, *TPX2*, *HMMR* and *CDC45*. The red boxes represent tumor tissues group, and the blue boxes represent normal tissue group. *p < 0.001. TCGA, The Cancer Genome Atlas, LIHC, liver hepatocellular carcinoma, TPM, Trans Per Million.

The top 10 hub genes with high degree identified by using CytoHubba, included *CDK1* (Cyclin-dependent kinase 1), *CCNB1* (cyclin-B1), *AURKA* (Aurora kinase A), *CCNA2* (Cyclin-A2), *KIF11* (kinesin family member 11), *BUB1B* (mitotic checkpoint serine/threonine kinase B), *TOP2A* (DNA topoisomerase II alpha), *TPX2* (Xenopus kinesin-like protein 2), *HMMR* (Hyaluronan mediated motility receptor) and *CDC45* (cell division cycle 45) (Figures 6A,B and Table 2). In addition, the selected hub genes were highly expressed in HCC tumor tissues compared with normal tissues of the TCGA dataset in GEPIA (Figures 7A–J), which is consistent with our results.

**TABLE 2:** The top 10 genes with the highest degree between HCC tissues and normal liver tissues.

Gene	Description	Degree	Related signal pathway	Up/down
CDK1	cyclin-dependent kinase 1	88	Cell cycle, p53 signaling pathway, Oocyte meiosis	Up
CCNB1	cyclin-B1	86	Cell cycle, p53 signaling pathway, Oocyte meiosis	Up
AURKA	Aurora kinase A	83	Oocyte meiosis	Up
CCNA2	cyclin-A2	82	Cell cycle	Up
KIF11	kinesin family member 11	82	Not mention	Up
BUB1B	mitotic checkpoint serine/threonine kinase B	81	Cell cycle	Up
TOP2A	DNA topoisomerase II alpha	81	Not mention	Up
TPX2	Xenopus kinesin-like protein 2	80	Unknown	Up
HMMR	Hyaluronan mediated motility receptor	80	Not mention	Up
CDC45	cell division cycle 45	79	Cell cycle	Up

HCC, hepatocellular carcinoma.

**FIGURE 8:**
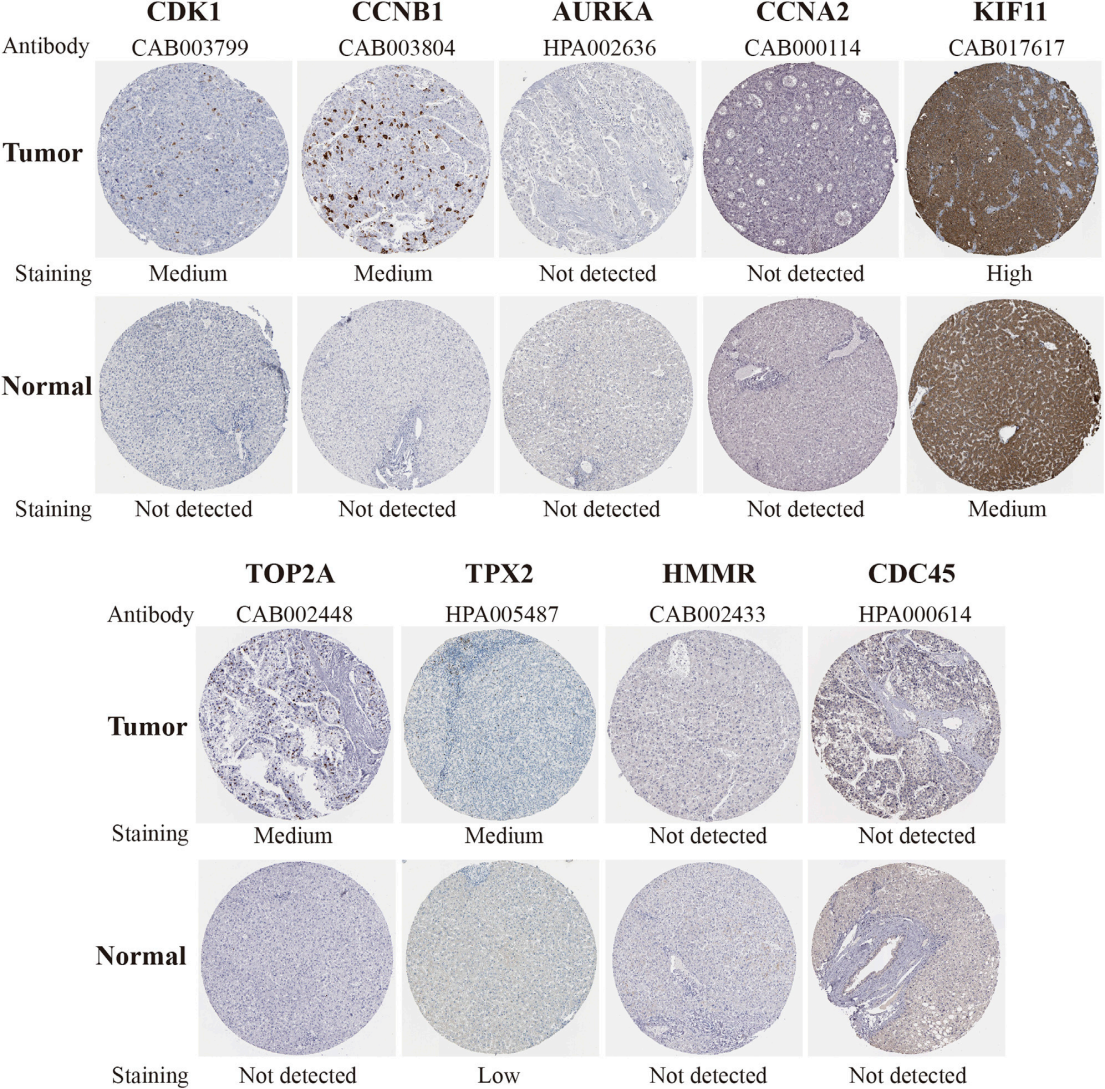
Immunohistochemical staining of protein level of candidate genes (*CDK1*, *CCNB1*, *AURKA*, *CCNA2*, *KIF11*, *TOP2A*, *TPX2*, *HMMR* and *CDC45*) in normal tissues and HCC tissues in the HPA database. The result of *BUB1B* was missing. HCC, hepatocellular carcinoma, HPA, Human Protein Atlas.

To further explore the hub genes protein expression in HCC, we analyzed immunohistochemistry staining images about *CDK1* (five samples), *CCNB1*(seven samples), *AURKA* (seven samples), *CCNA2*(six samples), *KIF11*(6 samples), *BUB1B*(none), *TOP2A* (7 samples), *TPX2* (6 samples), *HMMR* (3 samples) and *CDC45* (8 samples) in HCC tissues and normal tissues from the HPA(Figure 8). The result showed that the protein expression level of *CDK1* and *TOP2A* was negative in normal tissues and positive in HCC tissues. Moreover, the low protein expression levels of *TPX2* were revealed in normal liver tissues, while low (1/6) and medium (4/6) even none (1/6) protein expression levels were observed in liver cancer tissues. Besides, the protein expression level of *CCNA2* was negative in normal tissues and half of HCC samples (3/6). *CCNB1* proteins were not expressed in normal tissues, while medium protein expressions of *CCNB1* were expressed in most HCC samples (5/7). Also, medium protein expressions of *KIF11* were observed in normal breast tissues, while medium (3/6) and high (3/6) protein expressions in HCC tissues. Furthermore, *AURKA*, *HMMR*, and *CDC45* proteins were not expressed in normal and most HCC samples (4/7, 2/3, 6/8). In summary, compared with normal tissue, the above results indicated that translational expression levels of *CDK1* and *TOP2A* were overexpressed in HCC.

**FIGURE 9:**
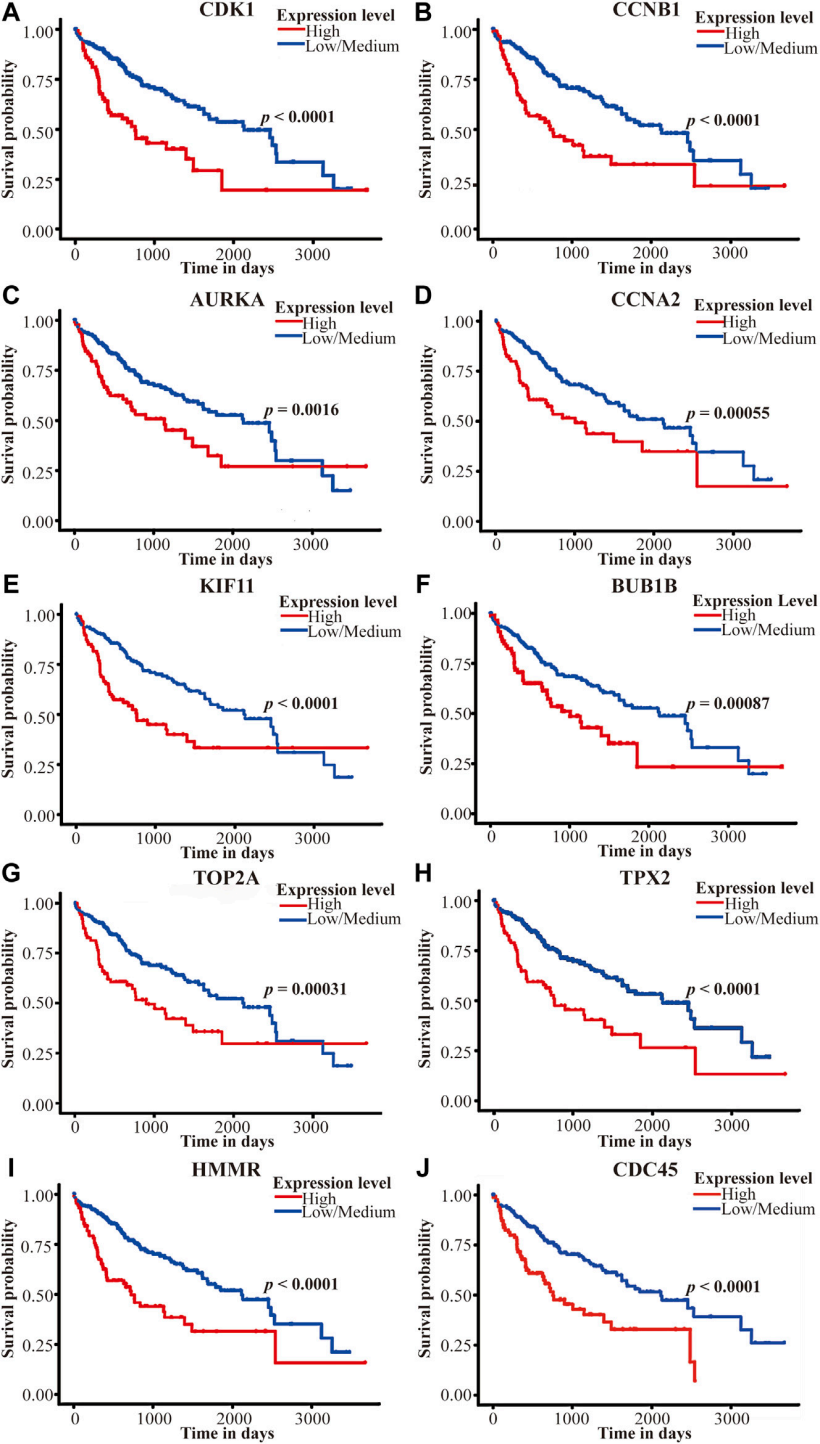
Survival analysis of the top 10 hub genes on the TCGA LIHC dataset in the UALCAN database. **(A–J)** represent *CDK1*, *CCNB1*, *AURKA*, *CCNA2*, *KIF11*, *BUB1B*, *TOP2A*, *TPX2*, *HMMR*, and *CDC45*. The red line represents the high-expression group, and the blue line represents the low/medium-expression group. *p* Value <0.05 was considered statistically significant. TCGA, The Cancer Genome Atlas, LIHC, liver hepatocellular carcinoma.

As is shown in Figures 9A–J, Over-expression of *CDK1* (*p* < 0.0001), *CCNB1* (*p* < 0.0001), *AURKA* (*p* = 0.0016), *CCNA2* (*p* = 0.00055), *KIF11* (*p* < 0.0001), *BUB1B* (*p* = 0.00087), *TOP2A* (*p* = 0.00031), *TPX2* (*p* < 0.0001), *HMMR* (*p* < 0.0001) and *CDC45* (*p* < 0.0001) was associated with poor overall survival (OS) among TCGA HCC patients in UALCAN. Furthermore, we conducted a subgroup analysis of HCC patients by ethnicity using Kaplan-Meier Plotter. In general, high expression of all ten genes was associated with poor prognosis, which is consistent with previous results. In Asian cohort, Over-expression of *CDK1* (hazard ratio [HR], 4.95; *p* = 4.7E-08), *CCNB1* (HR, 7.09; *p* = 6.3E-09, *AURKA* (HR, 4.5; *p* = 9.6E-07), *CCNA2* (HR, 5.27; *p* = 7.3E-07), *KIF11* (HR, 4.47; *p* = 1.3E-07), *BUB1B* (HR, 4.85; *p* = 2.4E-08), *TOP2A* (HR, 5.07; *p* = 7.5E-09), *TPX2* (HR, 5.95; *p* = 1.5E-10), *HMMR* (HR, 4.72; *p* = 2.9E-07) and *CDC45* (HR, 3.94; *p* = 1.6E-06) was associated with poor OS. Significantly, compared with White/Caucasian cohort, overexpression of hub genes in the Asian cohort predicted poorer survival outcomes (Table 3). This shows the potential of these genes as prognostic markers for HCC in Asia (including China).

**TABLE 3:** Survival for the ten hub genes in the Asian and White/Caucasian HCC cohorts from TCGA in Kaplan-Meier Plotter.

Gene	All (*N* = 364)		White (*N* = 184)		Asian (*N* = 158)	
	HR (95%CI)	*p*.Value	HR (95%CI)	*p*.Value	HR (95%CI)	*p*.Value
CDK1	2.15 (1.52–3.06)	1.1E–05	1.62 (1.01–2.59)	0.042	4.95 (2.63–9.32)	4.7E–08
CCNB1	2.34 (1.55–3.54)	3.4E–05	1.8 (1.1–2.94)	0.018	7.09 (3.29–15.29)	6.3E–09
AURKA	1.77 (1.25–2.5)	0.0011	1.34 (0.84–2.15)	0.22	4.5 (2.33–8.66)	9.6E–07
CCNA2	1.91 (1.36–2.72)	0.00018	1.9 (1.02–3.52)	0.039	5.27 (2.53–10.98)	7.3E–07
KIF11	2.02 (1.42–2.85)	5.5E–05	1.77 (1.11–2.82)	0.014	4.47 (2.44–8.2)	1.3E–07
BUB1B	2.01 (1.42–2.86)	6.6E–05	1.73 (1.01–2.96)	0.042	4.85 (2.64–8.92)	2.4E–08
TOP2A	1.99 (1.39–2.86)	0.00012	2.09 (1.17–3.76)	0.012	5.07 (2.76–9.33)	7.5E–09
TPX2	2.29 (1.62–3.24)	1.4E–06	2.54 (1.34–4.84)	0.0032	5.95 (3.21–11.02)	1.5E–10
HMMR	2.29 (1.62–3.24)	1.3E–06	2.43 (1.45–4.08)	0.00055	4.72 (2.46–9.05)	2.9E–07
CDC45	2.23 (1.51–3.28)	3.4E–05	1.59 (0.99–2.55)	0.053	3.94 (2.16–7.19)	1.6E–06

HCC, hepatocellular carcinoma; TCGA, The Cancer Genome Atlas; HR, Hazard Ratio; CI, confidence interval.

## Discussion

In the present study, A total of 293 common DEGs, including 103 up-regulated genes and 190 down-regulated genes, were identified in HCC tissues compared with normal hepatic tissues. Interestingly, as shown in the PPI network, most of the genes with higher connectivity were up-regulated genes, which were mainly enriched in cell cycle, cell division, and mitotic nuclear division. It suggested that common DEGs participate in the proliferation and division of HCC cells. The common DEGs and subnetworks were associated with signaling pathways such as metabolic pathways and cell cycle. Under the regulation of various carcinogenic pathways, cancer cells undergo adaptive metabolic reprogramming to maintain a specific metabolic state that supports their uncontrolled proliferation [20]. The latest research first used integrated proteogenomic characterization of paired tumor and adjacent liver tissues to reveal liver-specific metabolic reprogramming in HBV-related HCC [21]. Also, given the evidence that the epidemics of obesity, diabetes, and metabolic syndrome were considered as contributory factors to the occurrence of HCC [1, 5], the changes in metabolic pathways are not only the result of the progression of HCC but also may engage in the development of HCC. The recovery of abnormal metabolism provides a new idea for prevention, diagnosis, and treatment of HCC in China. The 10 hub genes were identified in the PPI network, including *CDK1*, *CCNB1*, *AURKA*, *CCNA2*, *KIF11*, *BUB1B*, *TOP2A*, *TPX2*, *HMMR* and *CDC45*. Although the critical genes screened are not the same in many earlier reports, these studies with similar results show that cell cycle and metabolic pathways play an essential role in the occurrence and development of liver cancer [22–24].

Moreover, all of the hub genes’ over-expression was related to poor prognosis in the TCGA database. Interestingly, compared with White group, the Asian group’s overexpression predicted poorer survival outcomes in Kaplan-Meier Plotter. This shows the potential of these genes as prognostic markers for HCC in Asia (including China). This may contribute to discovering biomarkers and drug targets for HCC in China that guide clinical practice and benefit patients.

It is widely believed that the cell cycle is closely linked with the advancement of cancers, while the disruption of the cell cycle is a characteristic of tumor cells. In this study, thirteen common DEGs containing half of ten hub genes, including *CCNB1*, *CDK1*, *CDC45*, *BUB1B* and *CCNA2*, enriched in the cell cycle. This result suggests that cell cycle plays a vital role in the development of HCC and provides new targets for identifying serological markers and therapies of HCC in China. Cyclins and cyclin-dependent protein kinases (CDKs) are important regulators for cell cycle progression [25]. *CCNB1*, usually called cyclin B1, is a key regulator of G2/M in the cell cycle [26]. Some studies have found that *CCNB1* expression is increased in different types of cancer, such as breast cancer [27] and gastric cancer [28]. *CDK1* is a member of serine/threonine protein kinases, which forms a complex with *CCNB1* to regulate the mitotic process and maintain the mitotic state [29]. It has been reported that *CDK1* is not only overexpressed in diffuse large B-cell lymphoma and melanoma but also highly expressed in colorectal cancer and prostate cancer [30]. Previous studies have shown that *CDK1/CCNB1* inhibits the p53 signaling pathway and regulate the development of HCC [8]. An *in vitro* study demonstrated that HBV X protein (HBx) induces G2/M phase arrest and apoptosis through continuous activation of *CDK1/CCNB1* kinase [31]. Other studies reported that both *CCNB1* and *CDK1* are overexpressed in HBV-related HCC tissues and are associated with poor survival [26].

*CCNA2*, usually called cyclin A2, binds and activates cyclin-dependent kinase 2 and promotes transition through G1/S and G2/M in the cell cycle. Some studies have implied that *CCNA2* is overexpressed in many types of cancers [32]. Other studies have revealed that *CCNA2* is overexpressed in HCC and may be relevant to poor prognosis [33], supporting our results. HBV integration is common in HBV-related HCC and may play an important role in the occurrence and development of HCC. In 1990, the study of Wang *et al.* first reported the integration of *CCNA2* gene and hepatitis B virus in HCC [34]. Recent studies have demonstrated that adeno-associated virus type 2 (AAV2) infection induces insertion mutations in tumors. *CCNA2*, as one of the insertion target genes of AAV2, is overexpressed in HCC, promoting cell cycle progression and showing its potential carcinogenic function [35]. Recently, Bayard *et al.* firstly described the recurrent fusion of the *CCNA2* gene in the non-cirrhotic liver cancer genome, which leads to oncogene activation by truncating a regulatory N-terminal domain [36].

*BUB1B* encodes a kinase involved in spindle checkpoint function and plays a role in delaying the onset of anaphase and ensuring proper chromosome segregation. Previous studies discovered that over-expression of *BUB1B* in tumor tissues predicts a poor prognosis of pancreatic ductal adenocarcinoma and adrenal carcinoma, while the low expression of *BUB1B* is associated with poor survival in patients with colon adenocarcinoma and lung cancer [26]. The up-regulation of *BUB1B* in tumor tissues of patients with HCC predicts poor OS and relapse-free survival (RFS) [37], which is consistent with our results. Nevertheless, its specific role in the development of HCC is still not completely clear, and further experiments are necessary.

*CDC45* (cell division cycle 45) plays a vital role in the initiation and extension of DNA replication in eukaryotic chromosomes [38]. The expression of *CDC45* increased in tongue squamous cell carcinomas, and its level was positively correlated with grades of precancerous lesions in epithelial dysplasia [39]. Recently, the overexpression of *CDC45* was found to predict poor prognosis in Asian HCC and HBV-related HCC [40, 41], similar to our research results.

In addition to *CCNB1*, *CDK1*, *CDC45*, *BUB1B,* and *CCNA2*, we also identified five hub genes in Chinese liver cancer, namely *AURKA*, *KIF11*, *TOP2A*, *TPX2,* and *HMMR*, which play a crucial role in regulating the cell cycle. *AURKA* is a cell cycle-regulated kinase that appears to be involved in microtubule formation and/or stabilization at the spindle pole during chromosome segregation. *AURKA* plays multiple roles in regulating cancer development, while its oncogenic roles might vary in different types of cancer. In the majority of solid tumors, *AURKA* works mainly through overriding cell cycle checkpoints and promoting cell cycle progression [42]. Chen *et al.* found that *AURKA* is up-regulated in HCC tissues, which is associated with pathological stage and distant metastasis [43]. Interestingly, *AURKA* is involved in tumor metastasis after radiotherapy for HCC. This is might because *AURKA* enhances the radiation resistance of HCC by activating the NF-κB signaling pathway [44].

*KIF11* encodes a motor protein belonging to the kinesin-like protein family, which is involved in various kinds of spindle dynamics. Previous studies have discovered over-expression of *KIF11* in a variety of cancers and suggested poor survival, while another study found that chromosome instability caused by *KIF11* silencing or inhibition may contribute to the development of cancers [45]. A study showed that *KIF11* overexpression was significantly correlated with HCC progression and prognosis in the TCGA database [46], which is consistent with our results. However, it is necessary to further investigate the function of *KIF11* and its exact mechanism in HCC.

*TOP2A* encodes a DNA topoisomerase, an enzyme that controls and alters the topologic states of DNA during transcription. Among all forms of topoisomerase, *TOP2A* is mainly involved in cell proliferation and overexpressed in a variety of cancers, and its overexpression causes the poor prognosis of these malignant tumors. In this study, *TOP2A* was found to be overexpressed in HCC, and its expression levels are positively correlated with poor prognosis, which was consistent with previous research [47]. Previous studies also revealed that TOP2A expression level was closely related to histological grade, microvascular invasion, and early onset. Furthermore, *TOP2A* was overexpressed in HBV-related HCC, which has close association with serum AFP [48].

*TPX2* has been considered as a critical factor in mitosis and spindle assembly due to the Ran-regulated microtubule-associated protein properties and its control of the Aurora-A kinase [49]. It has been reported that *TPX2* is overexpressed in many types of cancer, which is correlated with poor prognosis [50]. Our study found that overexpression of *TPX2* predicted a poor prognosis of patients with HCC in China. Previous clinical studies have shown that the expression of *TPX2* in liver cancer tissue is significantly related to tumor-node-metastasis stage, tumor number, differentiation, and stage [51]. Above all, *TPX2* could be a novel prognostic biomarker and a potential therapeutic target for HCC.

*HMMR*, also known as *RHAMM*, is one of the few known hyaluronan receptors. However, a recent review indicated that *HMMR* encodes evolutionarily conserved homeostasis, mitosis, and meiosis regulator instead of a hyaluronan receptor [52]. Additionally, *HMMR* is decreased in most healthy tissues but increased in hyperplastic tissues. It has been reported that *HMMR* is overexpressed in HCC tissues compared with normal tissues, and its level is associated with poor prognosis [53], which confirms our results. However, further laboratory experiments are needed to investigate the importance of *HMMR* in the development of HCC.

Prior to our study, there has been some research on bioinformatics analysis of key genes in HCC [26, 48, 53]. Nevertheless, our study still has several obvious advantages: Firstly, the datasets we selected are from different cities in China. Therefore, the identified genes have unique guiding significance for the early diagnosis and precise treatment of patients with HCC in China. Secondly, the microarray datasets were searched from January 1st, 2016 to October 30th, 2019, to eliminate the errors caused by the imbalance of high-throughput sequencing technology due to the results of some outdated datasets are relatively inaccurate. Thirdly, before identifying DEGs, each dataset’s sample was normalized, which enables accurate comparisons of expression levels between and within samples in this study. Finally, we used the TCGA, HPA, and other databases to verify the results obtained from the GEO database, and the combined use of those databases made the results more convincing than a single database. Unfortunately, our research still has the following deficiencies. Firstly, the four datasets we selected contain only 144 samples and cover four big densely populated cities in China. Secondly, our results were only based on the analysis of GEO, TCGA, and other public databases and have not been verified by molecular biology experiments. Thirdly, our study is an integrated analysis of HCC from China, without further subgroup analysis according to different pathogenesis and tumor types. Therefore, in further study, we will enlarge samples and supplement more multi-center clinical data and add some molecular biology experiments if possible.

## Conclusion

In summary, this study firstly screened out common DEGs and signaling pathways involved in the occurrence and development of HCC in China, on the basis of integrated bioinformatics analysis. In addition, the present study opened up new horizons for the specific etiology and molecular mechanisms of HCC and provided candidate biomarkers and new therapeutic targets for HCC in China.

## Data Availability

The datasets presented in this study can be found in online repositories. The names of the repository/repositories and accession number(s) can be found in the article/Supplementary Material.
