# Supplementary material for: Bioinformatics Analysis of Candidate Genes and Pathways Related to Hepatocellular Carcinoma in China: A Study Based on Public Databases
Source: Pathol Oncol Res. 2021 Mar 26;27:588532. doi: 10.3389/pore.2021.588532 (PMC8262246; doi:10.3389/pore.2021.588532)
Supplement: Supplementary file 1 [file Table1.DOCX]

Supplementary Material

**Supplementary Table S1. The common up-regulated DEGs were identified among GSE84005, GSE84402, GSE101685, and GSE115018.**

| Gene names (103 common up-regulated DEGs) |
| --- |
| TOP2A, PRC1, KIF4A, FOXM1, KIF20A, UBE2T, CDKN3, DEPDC1, CCNB1, AURKA, KIF23, CDK1, ESM1, CENPF, MKI67, DLGAP5, EXO1, DTL, MELK, ASPM, NUSAP1, HJURP, FANCI, BUB1B, CDCA8, CHEK1, CENPL, MSH2, GINS1, KIF11, PBK, FAM83D, CDCA3, KIF14, STIL, PTTG1, RACGAP1, CCNA2, EZH2, TPX2, RFC4, ZWINT, CDCA5, TTK, RRM2, WDHD1, E2F8, CAP2, ITGA6, CCDC34, DEPDC1B, BIRC5, HMMR, FLVCR1, GMNN, FEN1, NEK2, SHCBP1, CDC45, KIF18A, MCM6, FIGNL1, SMC4, TRIP13, MCM2, SPAG5, PLVAP, HELLS, TDRKH, MDK, CCNE2, NUP155, BLM, SPC25, ATAD2, NCAPG2, KIF15, KNTC1, HSPB1, GPC3, CEP55, OIP5, RAD51AP1, MCM3, CENPK, NEIL3, SKA1, TUBG1, PRIM1, CKS2, B3GNT5, BARD1, BRIP1, IGF2BP3, ENAH, LAPTM4B, TYMS, CLGN, G6PD, LMNB1, SERPINI1, RBM24, EIF5A2 |

DEGs, differentially expressed genes.

**Supplementary Table S2. The common down-regulated DEGs were identified among GSE84005, GSE84402, GSE101685, and GSE115018.**

| Gene names (190 common down-regulated DEGs) |
| --- |
| OIT3, STAB2, CLEC4G, CRHBP, FCN3, BMPER, CLEC1B, MARC, PTH1R, ECM1, CFP, VIPR1, CCBE1, HHIP, GPM6A, COLEC10, CYP1A2, CETP, PDE2A, CXCL14, LYVE1, TIMD4, RSPO3, NPY1R, PLAC8, GNA14, F8, CDHR2, CYP39A1, MT1H, AADAT, MT1M, NGFR, LIFR, PAMR1, EHD3, SRPX, LILRB5, ANKRD55, INMT, MAN1C1, CD5L, BCO2, CNDP1, RBMS3, UROC1, PRKAR2B, HAMP, HGF, APOF, CXCL12, SLC25A47, STEAP4, GSTZ1, COLEC11, FOS, MYCT1, DIRAS3, FXYD6, TFPI2, ACSM3, RND3, C7, EGR1, FOSB, SLCO1B3, RNF125, CBR4, ZFPM2, MRO, MT1G, EDNRB, CCDC3, PROZ, CYP26A1, GCGR, MFAP4, GGT5, MS4A6A, STARD5, NTN4, OLFML3, DCN, AXL, FTCD, ACADL, CPEB3, C9, SLC9B2, PHYHD1, MASP1, CD163, GADD45B, GABARAPL1, SOCS2, CIDEB, PLSCR4, CCL21, CSRNP1, MFSD2A, CYP2B6, GHR, IGFALS, LPA, C1orf162, THRSP, EPHX2, FYN, LDHD, KMO, PCK1, IGFBP3, NR3C2, OGDHL, FAT4, HCLS1, TM6SF2, HAO2, AKR1D1, ANK3, GYS2, F11, HBB, CFI, GCH1, IYD, IDO2, SHBG, DHODH, GPT2, GIMAP7, BCHE, BGN, ADH4, IGF1, ALDH2, GZMK, CYP4V2, HSD17B2, ADH1B, AKR7A3, CTSO, NNMT, ACSM5, S100A12, MT1X, MASP2, GBA3, FBP1, SLC22A1, LY6E, PON3, C6, KCND3, PZP, ASPN, CLRN3, SATB1, PGLYRP2, CYP2A7, PRELP, AR, DNAJC12, CYP2E1, CXCL2, ASPA, MAT1A, CRISPLD2, TDO2, SLC25A15, CYP2C19, CYP8B1, F9, ADH1C, C8B, MME, DTX1, EXPH5, CFHR4, SLCO1B1, BBOX1, ANGPTL3, PLG, DUSP1, SLC17A2, AVPR1A, SLC46A3, TPD52L1, SLC28A1, SLC1A1 |

DEGs, differentially expressed genes.

**Supplementary Table S3. Top 15 GO enrichment terms of common DEGs.**

| Category | Term | Description | Count | P.Value |
| --- | --- | --- | --- | --- |
| GO: BP | GO:0055114 | oxidation-reduction process | 28 | 1.89E-06 |
| GO: BP | GO:0051301 | cell division | 27 | 1.61E-10 |
| GO: BP | GO:0007067 | mitotic nuclear division | 24 | 2.33E-11 |
| GO: BP | GO:0008284 | positive regulation of cell proliferation | 17 | 0.004768435 |
| GO: BP | GO:0006508 | proteolysis | 17 | 0.008953802 |
| GO: CC | GO:0005829 | cytosol | 80 | 5.03E-05 |
| GO: CC | GO:0005654 | nucleoplasm | 62 | 0.003697221 |
| GO: CC | GO:0070062 | extracellular exosome | 61 | 0.007085234 |
| GO: CC | GO:0005576 | extracellular region | 54 | 1.68E-07 |
| GO: CC | GO:0005615 | extracellular space | 34 | 0.007183043 |
| GO: MF | GO:0005515 | protein binding | 174 | 9.15E-05 |
| GO: MF | GO:0005524 | ATP binding | 37 | 0.010260113 |
| GO: MF | GO:0042803 | protein homodimerization activity | 23 | 0.003869197 |
| GO: MF | GO:0019901 | protein kinase binding | 16 | 0.001244678 |
| GO: MF | GO:0004252 | serine-type endopeptidase activity | 15 | 7.31E-05 |

GO, gene ontology. BP, biological process. CC, cellular component. MF, molecular function. DEGs, differentially expressed genes.

**Supplementary Table S4. Top 5 enriched KEGG pathway terms of common DEGs.**

| Category | Term | Description | Count | P.Value |
| --- | --- | --- | --- | --- |
| KEGG pathway | hsa01100 | Metabolic pathways | 42 | 0.001184106 |
| KEGG pathway | hsa04110 | Cell cycle | 13 | 1.10E-05 |
| KEGG pathway | hsa04610 | Complement and coagulation cascades | 11 | 1.59E-06 |
| KEGG pathway | hsa00380 | Tryptophan metabolism | 8 | 1.74E-05 |
| KEGG pathway | hsa04115 | p53 signaling pathway | 8 | 5.04E-04 |

KEGG, Kyoto Encyclopedia of Genes and Genomes. DEGs, differentially expressed genes.

**Supplementary Table S5. Information on three subnetworks screened from the PPI network.**

| Subnetwork | Score | Nodes | Edges | Genes |
| --- | --- | --- | --- | --- |
| 1 | 65.493 | 72 | 2325 | STIL, KIF14, KIF18A, BIRC5, CDCA8, OIP5, HJURP, ZWINT, TRIP13, MELK, PRIM1, RFC4, DTL, CCNB1, HELLS, TPX2, EZH2, CCNE2, BUB1B, NEK2, MCM2, AURKA, RAD51AP1, DLGAP5, CKS2, KIF11, MKI67, KIF20A, FAM83D, EXO1, E2F8, CDCA5, MCM3, CDCA3, PRC1, MCM6, SHCBP1, CDC45, CCNA2, CDK1, KIF23, RACGAP1, WDHD1, SKA1, TTK, ASPM, CENPF, DEPDC1, TYMS, KIF4A, ATAD2, FEN1, KIF15, CDKN3, HMMR, NUSAP1, PTTG1, PBK, FOXM1, SPC25, TOP2A, SMC4, NCAPG2, CEP55, KNTC1, SPAG5, GMNN, FANCI, UBE2T, CHEK1, DEPDC1B, RRM2 |
| 2 | 7 | 7 | 21 | F9, C6, ANGPTL3, F11, FTCD, C8B, PROZ |
| 3 | 5.6 | 6 | 14 | CYP2B6, CYP2E1, CYP2C19, SLCO1B1, G6PD, CYP1A2 |

PPI, protein-protein interaction.

**Supplementary Table S6. KEGG enrichment of genes in the top 3** **subnetworks.**

| Subnetwork | Term | Count | P Value | Genes |
| --- | --- | --- | --- | --- |
| subnetwork1 | hsa04110:Cell cycle | 12 | 2.56E-13 | CCNB1, CCNE2, CDK1, CDC45, TTK, BUB1B, CHEK1, MCM2, PTTG1, MCM3, CCNA2, MCM6 |
|  | hsa03030:DNA replication | 6 | 1.79E-07 | PRIM1, RFC4, MCM2, MCM3, FEN1, MCM6 |
|  | hsa04115:p53 signaling pathway | 5 | 1.05E-04 | CCNB1, CCNE2, CDK1, RRM2, CHEK1 |
|  | hsa04114:Oocyte meiosis | 5 | 7.30E-04 | CCNB1, CCNE2, CDK1, AURKA, PTTG1 |
|  | hsa05203:Viral carcinogenesis | 4 | 0.040972941 | CCNE2, CDK1, CHEK1, CCNA2 |
|  | hsa04914:Progesterone-mediated oocyte maturation | 3 | 0.042229271 | CCNB1, CDK1, CCNA2 |
| subnetwork2 | hsa04610:Complement and coagulation cascades | 4 | 3.84E-06 | F11, C8B, C6, F9 |
|  | hsa05020:Prion diseases | 2 | 0.019628472 | C8B, C6 |
| subnetwork3 | hsa00982:Drug metabolism - cytochrome P450 | 4 | 9.11E-06 | CYP2C19, CYP2B6, CYP2E1, CYP1A2 |
|  | hsa00591:Linoleic acid metabolism | 3 | 1.70E-04 | CYP2C19, CYP2E1, CYP1A2 |
|  | hsa00590:Arachidonic acid metabolism | 3 | 7.60E-04 | CYP2C19, CYP2B6, CYP2E1 |
|  | hsa00980:Metabolism of xenobiotics by cytochrome P450 | 3 | 0.001118017 | CYP2B6, CYP2E1, CYP1A2 |
|  | hsa05204:Chemical carcinogenesis | 3 | 0.001305716 | CYP2C19, CYP2E1, CYP1A2 |
|  | hsa01100:Metabolic pathways | 5 | 0.004216216 | G6PD, CYP2C19, CYP2B6, CYP2E1, CYP1A2 |
|  | hsa00140:Steroid hormone biosynthesis | 2 | 0.041464218 | CYP2E1, CYP1A2 |
|  | hsa00830:Retinol metabolism | 2 | 0.045673855 | CYP2B6, CYP1A2 |

KEGG, Kyoto Encyclopedia of Genes and Genomes.


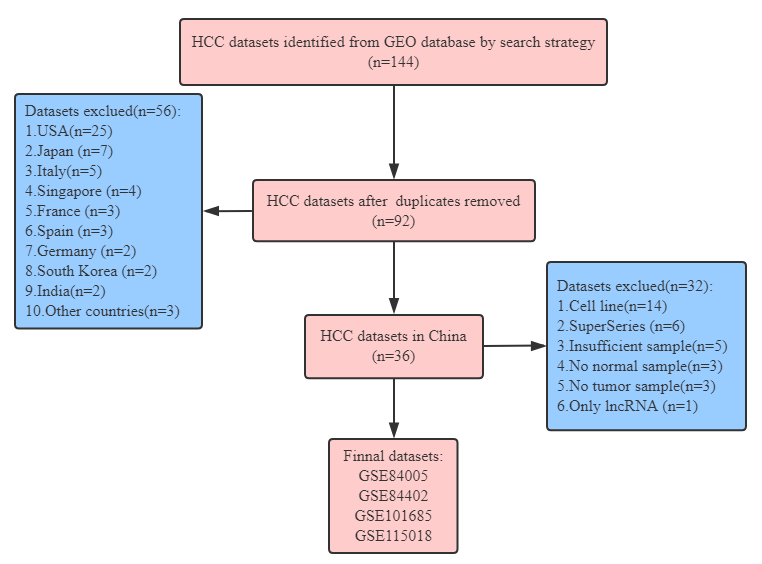


**Supplementary Figure S1. Flow chart for datasets selection.** GEO, Gene Expression Omnibus.


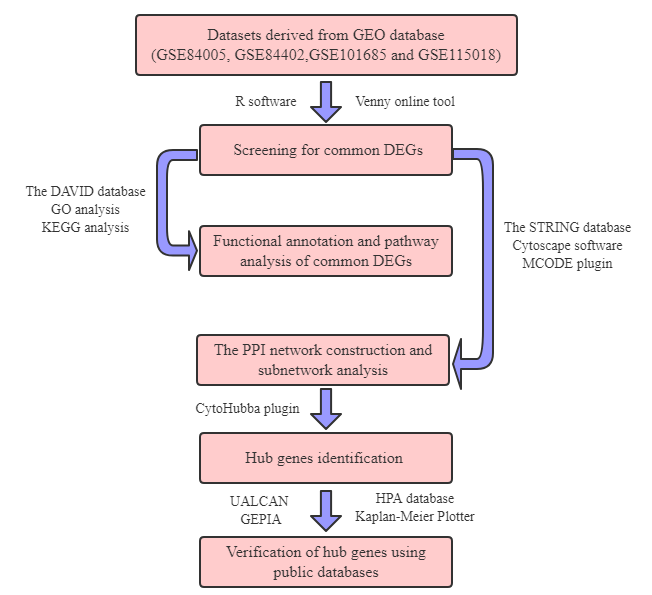


**Supplementary Figure S2. The process of identifying hub genes and pathways related to hepatocellular carcinoma in China.** GEO, Gene Expression Omnibus; DEGs, differentially expressed genes; DAVID, Database for Annotation, Visualization and Integrated Discovery; GO, Gene Ontology; KEGG, Kyoto Encyclopedia of Genes and Genomes; STRING, Search Tool for the Retrieval of Interacting Genes; PPI, protein-protein interaction; GEPIA, Gene Expression Profiling Interactive Analysis; HPA, Human Protein Atlas.


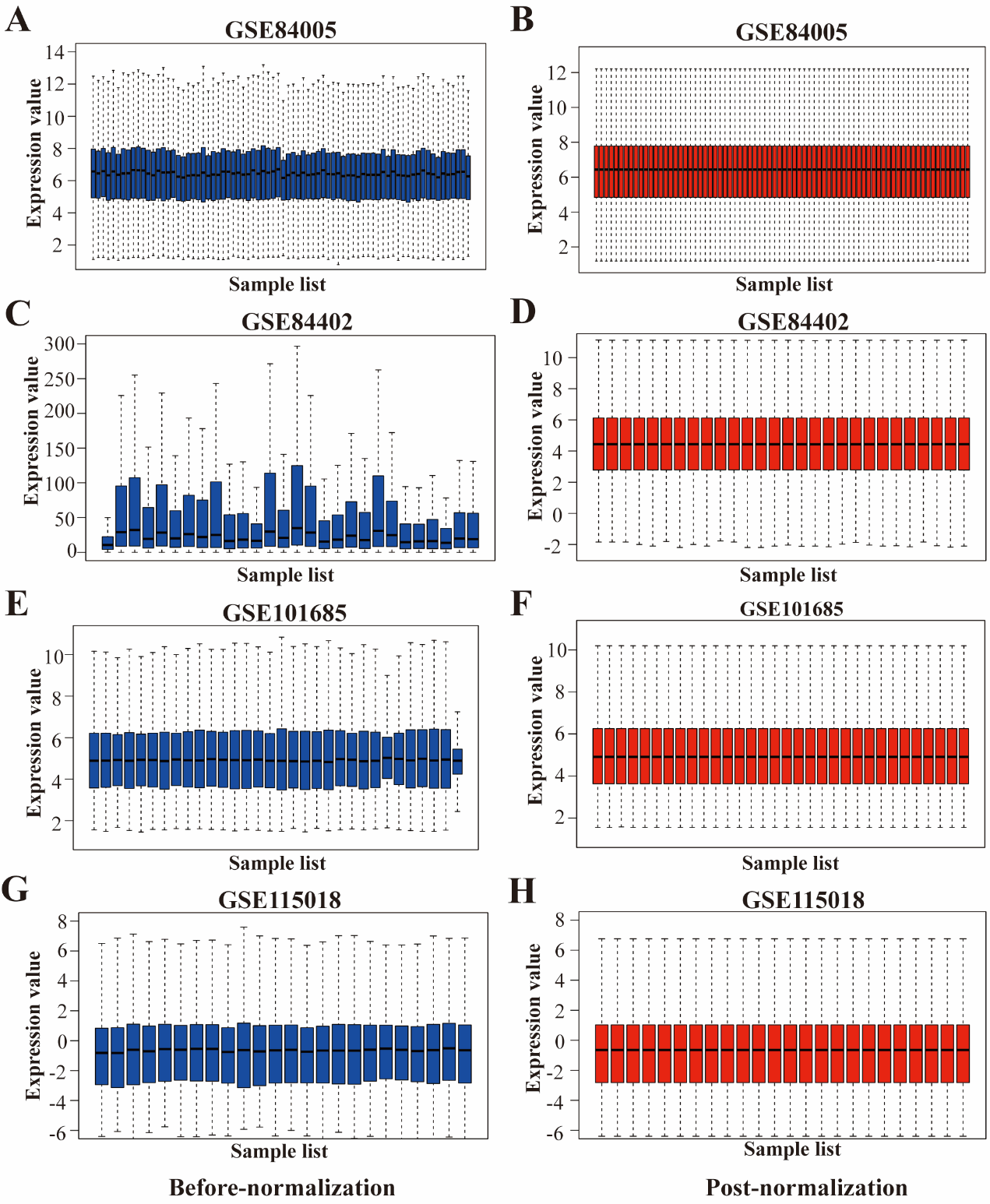


**Supplementary Figure S3. Normalization of gene expression matrices for each dataset.** (**A-B**), (**C-D**), (**E-F**), and (**G-H**) represent the normalization of the GSE84005, GSE84402, GSE101685, and GSE115018. Blue is pre-normalized data, and red is post- normalized data.


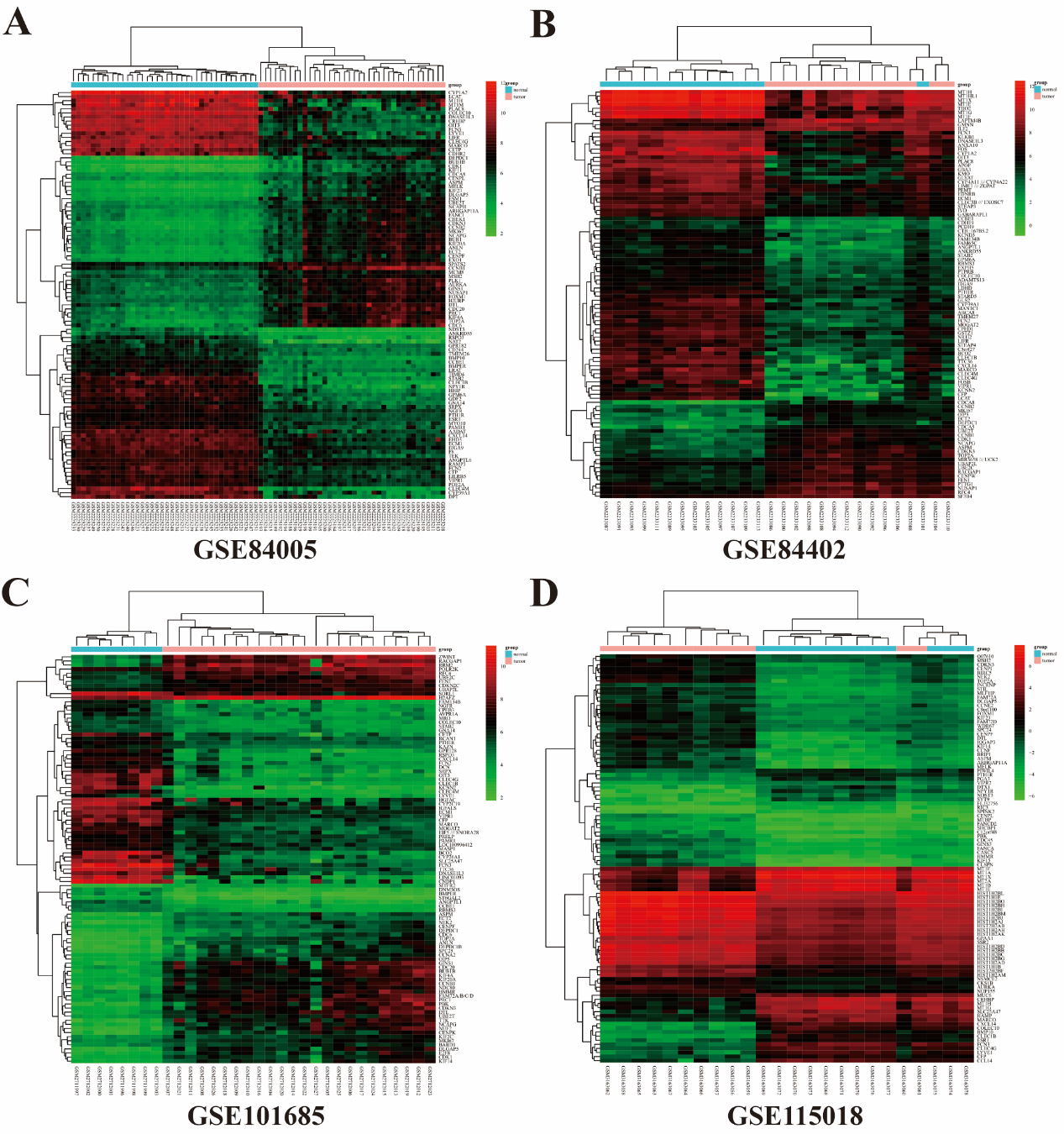


**Supplementary Figure S4. Heatmap of the top 100 DEGs expressions in each dataset.** (**A**)GSE84005, (**B**)GSE84402, (**C**)GSE101685 and (**D**)GSE115018. The abscissa represents the samples (including HCC tissue and normal tissue), the ordinate represents the gene name. The red (log FC > 0) represents up-regulated genes, the black (log FC = 0) represents genes with no significant difference, the green (log FC < 0) represents down-regulated genes. DEGs, differentially expressed genes.


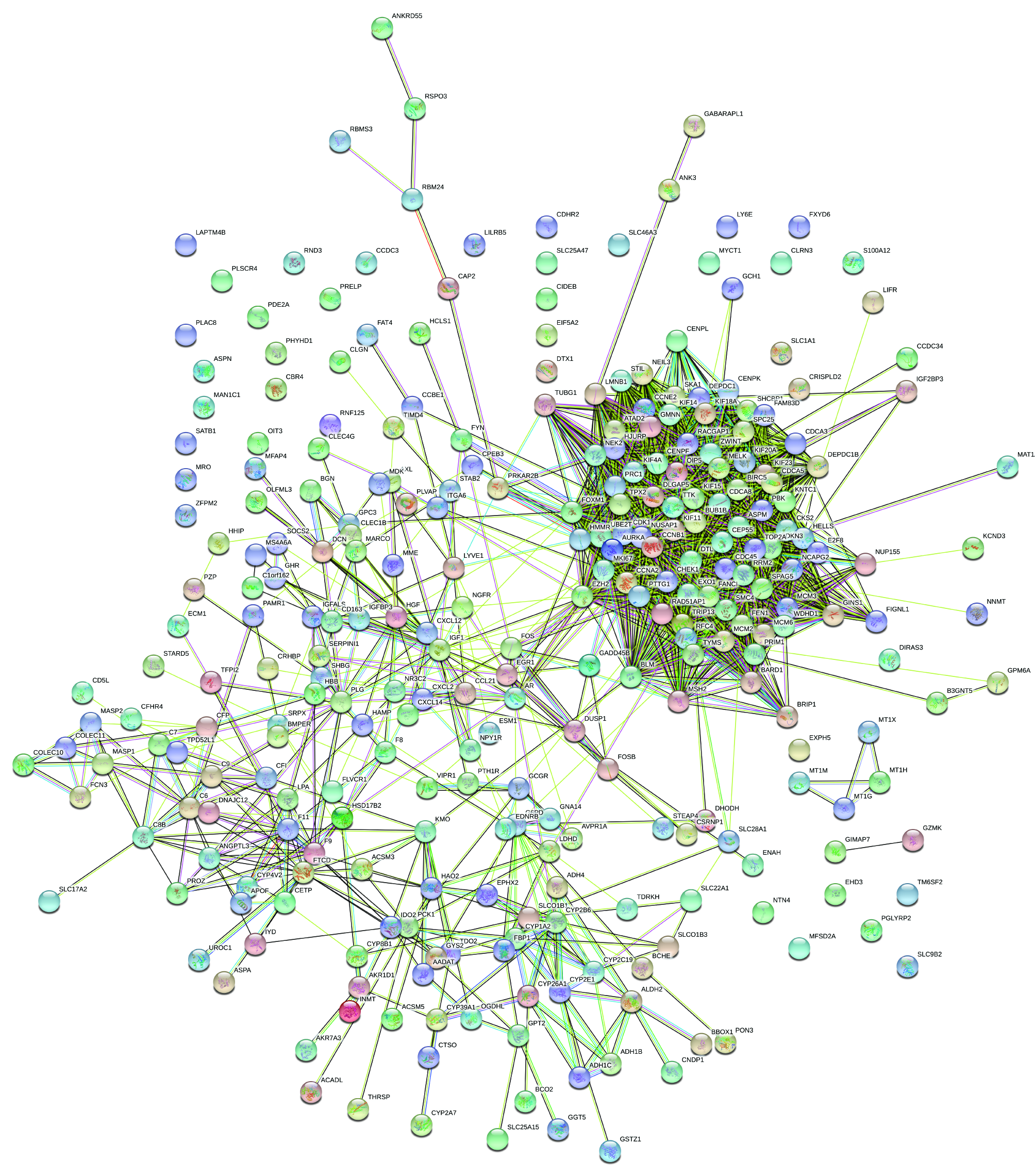


**Supplementary Figure S5. PPI network diagrams of common DEGs from the STRING online database.** PPI, protein-protein interaction. DEGs, differentially expressed genes. STRING, Search Tool for the Retrieval of Interacting Genes.


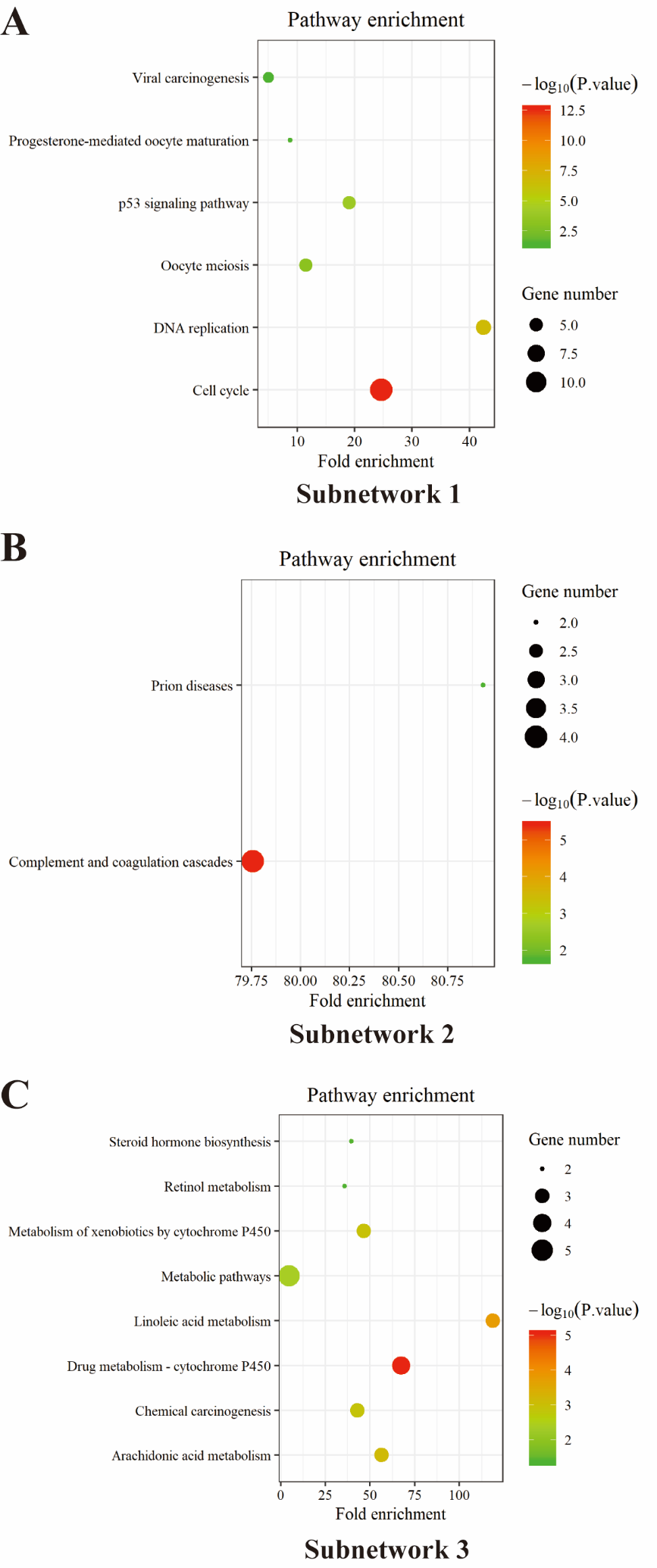


**Supplementary Figure S6. KEGG enrichment of genes in the subnetworks.** (**A**)subnetwork 1, (**B**)subnetwork 2, (**C**)subnetwork 3. The abscissa represents the fold enrichment; the ordinate represents the pathway terms. The bubble size represents the gene number enriched in this pathway, and the color of the bubble represents a statistical difference. KEGG, Kyoto Encyclopedia of Genes and Genomes.
